# Stellate Ganglion Block for the Management of Long COVID Symptoms: A Retrospective Cohort Study

**DOI:** 10.7759/cureus.88680

**Published:** 2025-07-24

**Authors:** Michael C Chiang, Kathryn M Satko, Christina Shin, Beau P Sperry, Meghan L Cabral, Zack Crockett, Richard Gao, Robert J Yong, Samuel P Ang, Stacey L Burns, Alexander J Kim

**Affiliations:** 1 Anesthesiology, Brigham and Women's Hospital, Harvard Medical School, Boston, USA; 2 Anesthesiology, University of Virginia, Charlottesville, USA; 3 Physical Medicine and Rehabilitation, Spaulding Rehabilitation Hospital, Harvard Medical School, Boston, USA; 4 Anesthesiology, Allegheny Health Network, Philadelphia, USA

**Keywords:** covid-19, long covid, post-acute sequelae of sars-cov-2 (pasc), stellate ganglion block (sgb), sympathetic hyperactivity

## Abstract

Introduction

The efficacy of stellate ganglion block (SGB) for managing post-acute sequelae of SARS-CoV-2 (PASC) remains an area of active exploration. In this study, we performed a retrospective analysis of PASC symptom improvements following SGB.

Methods

We performed a retrospective, survey-based cohort study at a single institution of patients with a history of PASC who underwent SGB at one of three different sites within Boston, MA, USA, between September 2022 and September 2024. The intervention was outpatient, office-based, with one or more unilateral SGBs.

Results

Fifty-two patients were included in the analysis. On average, patients underwent three SGB injections. Most patients reported improvement in PASC symptoms after SGB treatment. The most commonly reported symptoms that showed improvement were brain fog, fatigue, dizziness, and headache. A quarter of patients reported experiencing at least one adverse effect that was mostly short-lived.

Conclusion

SGB reduced the severity of many PASC symptoms, although there was high variability in the duration of symptom improvement. Further high-quality randomized clinical trials are warranted to investigate SGBs for the effective treatment of PASC.

## Introduction

Severe acute respiratory syndrome coronavirus 2 (SARS-CoV-2) has had a profound impact on public health, giving rise to a global pandemic [1]. Although many recover from the sequelae of an acute infection, a subset of patients continue to experience persistent symptoms beyond four weeks. This syndrome has been termed long COVID, post-COVID syndrome, or post-acute sequelae of SARS-CoV-2 (PASC) [2]. It has been estimated to affect 6.6% to 10.3% of infected patients [3]. Indeed, according to the Centers for Disease Control and Prevention (CDC), among adults who have had acute COVID infection at any point, 8.7% had active long COVID as of August 2024. Furthermore, this syndrome has been codified into the International Classification of Diseases (International Classification of Diseases, 10th Revision, Clinical Modification (ICD-10-CM)) [4].

PASC comprises a constellation of symptoms impacting multiple organ systems. The World Health Organization has provided a clinical definition with symptoms such as brain fog, headache, shortness of breath, post-exertional malaise, tachycardia, and fatigue. PASC has prompted extensive research into understanding and managing these lingering symptoms with a hypothesis that aberrant sympathetic hyperactivity or autonomic dysfunction may underlie the wide range of physiological impact [5-7]. Stellate ganglion blocks (SGBs) are performed for a variety of sympathetically mediated chronic pain syndromes and non-pain indications to modulate autonomic function and reduce sympathetic hyperactivity [8]. 

SGBs work by targeting the synapse of both preganglionic and postganglionic sympathetic fibers arising from the first thoracic segments of the sympathetic chain, which converge as the stellate ganglion. These sympathetic fibers provide innervation to the head, neck, upper extremities, and thoracic viscera. The wide range of SGB treatment indications includes complex regional pain syndrome of the upper extremities, peripheral vascular disease, hyperhidrosis, cardiac arrhythmias, and atypical chest pain [8-11]. In terms of cardiac arrhythmias, SGBs have clearly established use in targeting cardiac accelerator (sympathetic) fibers to effectively treat refractory ventricular tachycardia and ventricular fibrillation [12]. Additionally, SGBs have also been used to treat mood and headache disorders, particularly those relating to post-traumatic stress disorder (PTSD) and cluster headaches [13]. It is hypothesized that the constellation of symptoms comprising PASC is at least partially related to sympathetic and autonomic dysfunction, as well as possible mood disturbance. Accordingly, we hypothesized that SGBs would appropriately target and reduce the symptoms found in PASC.

We performed a retrospective analysis of the efficacy of SGB on the improvement of PASC symptoms for patients impacted by the syndrome. Our study aims to help further characterize the efficacy with a focus on delineating successfully treated symptoms and longitudinal durability. This retrospective survey study covers a two-year period of patients undergoing SGB for treatment of PASC.

## Materials and methods

We performed a retrospective cohort study without a control group at a single institution of patients with a history of PASC who underwent SGB at one of three different sites within Boston, MA, USA, between September 2022 and September 2024. The study was performed in accordance with the Mass General Brigham Institutional Review Board (IRB) (approval number: 2023P003108).

Study participants

All adult patients who underwent at least one SGB procedure with the intention of treating PASC at one of the included treatment facilities were included in this study. PASC was identified as new or worsening pre-defined symptoms persisting for at least four weeks after resolution of an acute COVID-19 infection, and which could not be attributed to any other condition. History of COVID-19 infection was determined by a positive polymerase chain reaction (PCR) test or antigen laboratory result, diagnosis by a medical provider, or patient report of a positive COVID-19 test with expected symptoms. All patients had previously been diagnosed with PASC by the referring medical provider and had undergone clinical investigation to rule out other non-COVID underlying conditions. After an evaluation at our pain management centers, patients were offered treatment consisting of consecutive left and right SGBs, a minimally invasive, office-based procedure. This procedure consists of the deposition of local anesthetic and/or steroid at the lower cervical sympathetic chain under ultrasound guidance and was performed by board-certified interventional pain physicians trained in this technique. Real-time ultrasound imaging was used to identify pertinent landmarks, including the C6 or C7 transverse process, thyroid gland, internal jugular vein, carotid artery, and longus colli muscle. The needle was advanced carefully in plane with the ultrasound probe such that the tip and medication deposition were into the plane ventral to the longus colli and dorsal to the carotid artery. The injectate composition and volume varied among providers. The vast majority included 6-10 milliliters of 0.25% bupivacaine. On only a few occasions, the local anesthetic in the injectate was 0.5% lidocaine. Approximately half of the injections were performed with the addition of a non-particulate corticosteroid (10 milligrams of dexamethasone). Contralateral SGB was offered one to two weeks later, and repeat ipsilateral SGB was offered two to three months later if the first procedure provided improvement of symptoms. Following completion of at least one SGB procedure, patients were provided with an electronic survey to assess outcomes, without a minimum amount of time required between SGB and survey completion. Patients who did not ultimately proceed with SGB intervention or did not complete the post-procedure survey were not included in our study.

Outcomes

Study outcomes were derived from a 54-item electronic survey sent to patients identified as having received one or more SGB for treatment of PASC (Appendix A). The survey was constructed after careful review of the existing literature of survey reports that assess patient outcomes, for which, at the time, no PASC-specific validated surveys existed. Questions on the diagnosis of COVID-19, the occurrence of PASC symptoms, and the severity of symptoms were derived from the CDC National Center for Health Statistics Household Pulse Survey. Patients were also asked to rate the severity of their PASC symptoms before and after SGB using a 10-point scale. Similar scales have been widely implemented in clinical and research settings [14-16]. Outcomes were also assessed using the Patient Global Impression of Change (PGIC) measure, which has been widely adopted in clinical trials for pain [16].

This survey collected patients’ baseline characteristics as well as information on patients’ specific PASC symptoms, symptom severity, and duration of illness. The following PASC symptoms were included in the survey: abnormal movements, headache, fatigue, dizziness, difficulty thinking or concentrating (brain fog), anxiety, chest pain, sleep disturbance, anosmia, palpitations, depression, issues with sexual desire or capacity, gastrointestinal symptoms, worsening of symptoms after mild physical or mental activity (post-exertional malaise), or increased thirst. Patients were invited to report additional symptoms under the category of “other.” From the completed surveys, additional reported symptoms included the following: dysautonomia, myalgia, orthostasis, shortness of breath, arthralgia, tinnitus, dysesthesia, dysgeusia, hair loss, nausea, parosmia, and unsteadiness. The surveys also collected information on the number of SGB treatments and outcomes. The perceived overall change in PASC symptoms was rated on a six-point scale from “much better,” “better,” “slightly better,” “no change,” “slightly worse,” to “much worse.” In addition to overall change, patients were further queried on specific symptom severity prior to and after their SGB treatment on a severity rating scale of 0-10, with 10 rated as markedly severe and debilitating. Additionally, the surveys collected reports of any adverse outcomes experienced after the SGB treatments. Horner’s syndrome is an expected finding of SGB and, therefore, was not considered an adverse effect. Patients received a short explanation of Horner’s syndrome and were asked to exclude its manifestations when reporting adverse effects post-SGB injection.

Statistical analysis

The responses of the patients were reported with the mean with standard deviation (SD) for numerical variables and count (percentage) for categorical variables. The median was reported for the severity of symptom ratings pre- and post-treatment. Analysis of descriptive data was performed using R (The R Core Team, R Foundation for Statistical Computing, Vienna, Austria) per the Brigham and Women’s Hospital Department of Anesthesiology research staff. All data were handled per Mass General Brigham Human Research Affairs Office regulations and practices. 

## Results

From September 2022 to September 2024, a total of 125 patients presented to the Brigham and Women’s Hospital Pain Management Centers with a diagnosis of PASC. One hundred and two patients underwent one or more ultrasound-guided SGB for treatment of their PASC symptoms. Twenty-three patients did not move forward with SGB due to a variety of reasons, including lack of insurance coverage, high out-of-pocket expense, necessity for further medical evaluation, or patient preference. Ultimately, 52 patients completed the survey and were included in this retrospective study.

The baseline characteristics of the 52 patients are presented in Table 1. The mean (SD) age of the patients was 45.8 (12.7) years. Thirty-three (63.5%) patients identified as female, and 43 (82.7%) patients identified as White. The mean (SD) duration of PASC symptoms prior to survey completion was 2.6 (0.9) years.

**Table 1:** Demographic information of the study group GED: General Educational Development

Parameters	n (%)	
Age, years (SD)	45.8 (12.7)	
Education		
Associate's degree	2 (3.8%)	
Bachelor's degree	20 (38.5%)	
Graduate or professional degree	20 (38.5%)	
High school diploma or GED	1 (1.9%)	
Professional certification and/or license	1 (1.9%)	
Some college	7 (13.5%)	
Some graduate school	1 (1.9%)	
Income		
$25,001 - $35,000	1 (1.9%)	
$35,001 - $50,000	3 (5.8%)	
$50,001 - $75,000	3 (5.8%)	
$75,001 - $100,000	10 (19.2%)	
Above $100,000	27 (51.9%)	
Below $15,000	3 (5.8%)	
I decline to answer	5 (9.6%)	
Race		
American Indian or Alaska Native	1 (1.9%)	
Asian	1 (1.9%)	
Hispanic, Latino, or Spanish	3 (5.8%)	
Middle Eastern or North African	1 (1.9%)	
White	43 (82.7%)	
None	2 (3.8%)	
Prefer not to answer	1 (1.9%)	
Gender		
Female	33 (63.5%)	
Male	19 (36.5%)	
Symptom duration, years (SD)	2.6 (0.9)	

The prevalence of all reported PASC symptoms is presented in Table 2. The symptoms most frequently reported in the survey were fatigue and brain fog (both 73.1%), and most patients reported both symptoms. Post-exertional malaise (48.1%), dizziness (34.6%), sleep disturbance (30.8%), and headache (30.8%) were also commonly reported. Twenty-one patients (40.4%) reported additional symptoms categorized as “other” (Appendix B). An overwhelming majority of patients (86.5%) reported that their symptoms significantly impacted their quality of life. 

**Table 2:** Prevalence of reported symptoms

Symptom	N (%)
Abnormal movements	1 (1.9%)
Headache	16 (30.8%)
Fatigue	38 (73.1%)
Dizziness	18 (34.6%)
Brain fog	38 (73.1%)
Anxiety	10 (19.2%)
Chest pain	10 (19.2%)
Sleep disturbance	16 (30.8%)
Anosmia	8 (15.4%)
Palpitations	12 (23.1%)
Depression	4 (7.7%)
Sexual dysfunction	2 (3.8%)
Other	21 (40.4%)
GI symptoms	6 (11.5%)
Post-exertional malaise	25 (48.1%)
Increased thirst	1 (1.9%)
Symptom severity	
Mild	1 (1.9%)
Moderate	6 (11.5%)
Severe	45 (86.5%)

Across the 52 patients, a total of 147 SGBs were performed. Twenty-five (48.1%) of the study patients received bilateral SGBs, performed at separate visits. Six (11.5%) patients received only one injection, and 13 (25%) patients received an SGB four or more times. The distribution of the number of SGBs performed in a single patient is reported in Table 3. There was a mean (SD) interval of 1.6 (0.9) years between PASC onset and first SGB treatment. 

**Table 3:** Number of SGBs per patient SGB: stellate ganglion block

Time to first injection, years (SD)	1.6 (0.9)
Injections per patient	n (%)
1	6 (11.5%)
2	25 (48.1%)
3	8 (15.4%)
≥ 4	13 (25.0%)
Total N	147
Average # per patient	3 (2)

Data on patient outcomes after SGB treatment were collected via a survey. We assessed the following dimensions of SGB treatment outcome: degree of overall improvement, duration of improvement, and change in individual symptom severity. Twenty-nine (55.8%) patients reported at least short-term improvement in their PASC symptoms after SGB treatment. Twelve patients (23.1%) reported symptom improvement for months, and seven (13.5%) reported persistent improvement up to the time of the survey. Eight patients reported improvement of limited duration-from just a few hours (5.8%) to several days (9.6%). Overall, 17 patients (32.7%) reported the greatest improvement after the first injection (Table 4).

**Table 4:** Effectiveness of SGB reported by patients. SGB: stellate ganglion block

Parameters	N (%)
Most helpful injection	
First	17 (32.7%)
Second	6 (11.5%)
Third	3 (5.8%)
Fourth	3 (5.8%)
Duration of improvement	
Hours	3 (5.8%)
Days	5 (9.6%)
Weeks	2 (3.8%)
Months	12 (23.1%)
Persistent improvement	7 (13.5%)
Rated change in symptoms	
Much better	11 (21.2%)
Better	9 (17.3%)
Slightly better	4 (7.7%)
No change	18 (34.6%)
Slightly worse	7 (13.5%)
Much worse	3 (5.8%)

Global change in symptom severity was reported on a six-point Likert scale. Twenty-four patients (46.2%) reported that their symptoms were “slightly better”, “better”, or “much better”. It should be noted that some patients who reported short-term symptom improvement (hours or days) reported no change on the Likert scale at the time of survey completion.

**Figure 1:**
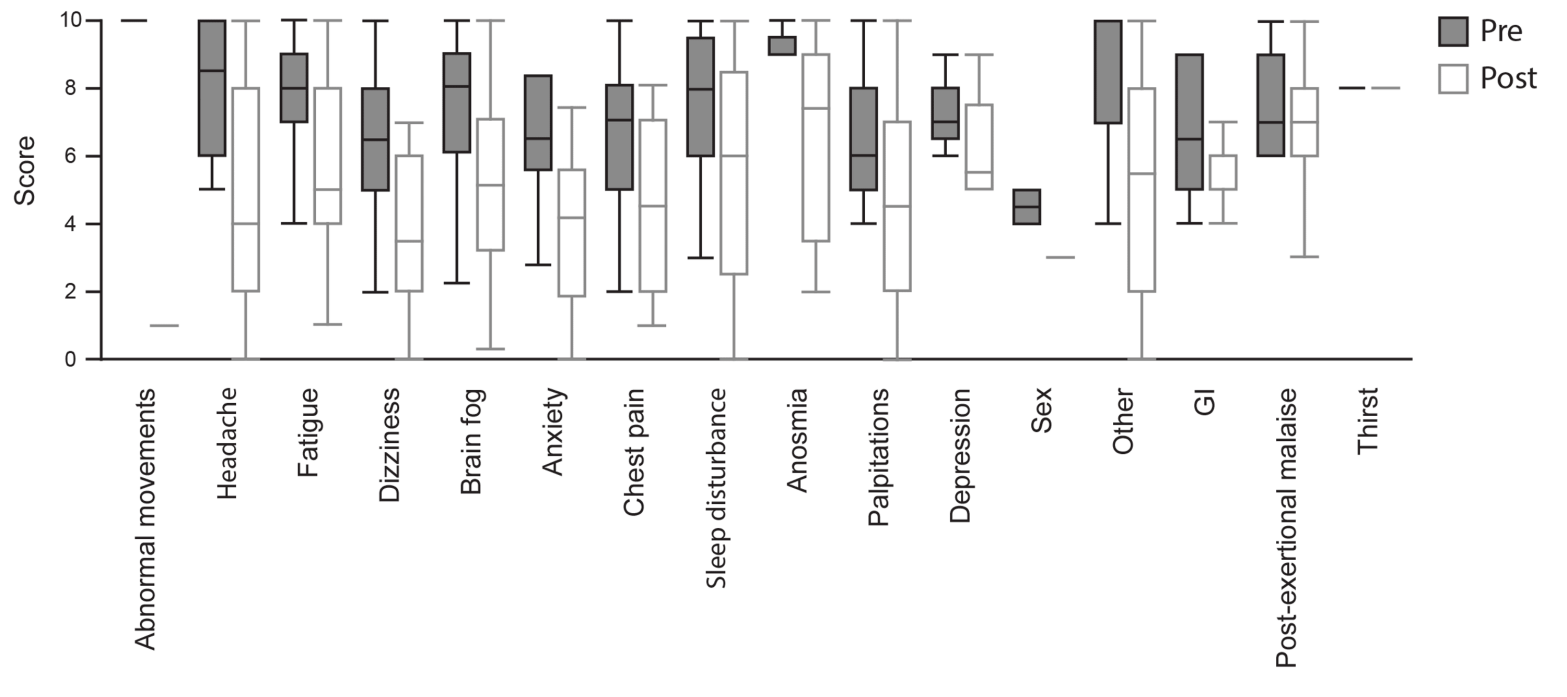
Pre- and post-SGB PASC symptom severity (top) Summary of patients' symptom severity before (gray) and after (white) SGB. Data are presented as median with quartiles and error bars in SD. SGB: stellate ganglion block; PASC: post-acute sequelae of SARS-CoV-2

The improvement in individual symptom severity was reported as the difference between median pre- and post-SGB scores (Table 5, Figure 1). The most commonly reported symptoms to show improvement were brain fog, fatigue, dizziness, and headache. The improvement in post-SGB symptom severity was by three, three, three, and 4.5 points, respectively. The greatest improvement (nine points) was seen in the symptom of abnormal movements, albeit this particular symptom was reported only in one patient. The least improvement was seen in GI symptoms (0.5 points) and post-exertional malaise (0 points). The highest percentage of patients reporting improvement of individual symptoms included those reporting abnormal movements (100%), sexual dysfunction (100%), headache (81.3%), anosmia (75%), anxiety (70%), and brain fog (65.8%).

**Table 5:** Change in PASC symptoms before and after SGBs Median scores of symptom severity before and after SGB. The number of patients reporting symptom improvement is shown on the right-most column. SGB: stellate ganglion block; PASC: post-acute sequelae of SARS-CoV-2

Symptoms	Pre	Post	Difference	N with improvement (%)
Abnormal movements	10	1	9	1 (100%)
Headache	8.5	4	4.5	13 (81.3%)
Fatigue	8	5	3	22 (57.9%)
Dizziness	6.5	3.5	3	10 (55.6%)
Brain fog	8	5	3	25 (65.8%)
Anxiety	7	4.5	2.5	7 (70%)
Chest pain	7	4.5	2.5	4 (40%)
Sleep disturbance	8	6	2	6 (37.5%)
Anosmia	9	7.5	1.5	6 (75%)
Palpitations	6	4.5	1.5	5 (41.7%)
Depression	7	5.5	1.5	2 (50%)
Sexual dysfunction	4.5	3	1.5	2 (100%)
Other	7	5.5	1.5	13 (61.9%)
GI symptoms	6.5	6	0.5	2 (33.3%)
Post-exertional Malaise	7	7	0	11 (44%)
Thirst	8	8	0	0 (0%)

Data on any adverse effects following SGB treatment were collected via a survey. Thirteen patients (25%) reported an adverse effect after receiving an SGB (Table 6). New-onset headaches and worsened post-exertional malaise were the most frequently reported (three patients). Additional reported adverse events included worsened fatigue, pain at the injection site, palpitations, increased startle, nausea, dizziness, hoarseness, restlessness, and shortness of breath. These events were reported to last from hours to months.

**Table 6:** Prevalence of adverse events Duration was reported on a scale of hours (less than 24 hours), days (between one and seven days), weeks (between one and four weeks), or months (> 4 weeks).

Adverse event	Frequency	Duration
New onset headache	3	Days
Worsened post-exertional malaise	3	Weeks - months
Worsened fatigue	2	Days - weeks
Pain at injection site	2	Days
Palpitations	2	Hours
Increased startle	1	Weeks
Nausea/dizziness	1	Weeks
Hoarseness	1	Hours
Restlessness	1	Weeks
Shortness of breath	1	Hours

## Discussion

The global prevalence of long COVID or PASC has been conservatively estimated at 65 million patients [17, 18]. From the 2022 National Health Interview Survey by the CDC, 36.6% of American adults reported ever having COVID-19, and 17.7% of those adults reported ever having long COVID. Among those, 48.5% reported having recovered. In addition, specific diagnostic details of PASC remain somewhat vague due to the high variability of presenting symptoms and lack of confirmatory laboratory or radiologic diagnosis. An NIH study did not find evidence of persistent viral infection, autoimmunity, or abnormal immune activation in those with PASC [19]. Another study showed that most patients with PASC do not show lesions on magnetic resonance imaging of the brain [20].

Overall, there is a substantial healthcare and societal burden. Despite the large total number of patients affected, no specific treatment options have been validated as the gold standard among medical professionals, nor is there a Food and Drug Administration (FDA)-approved treatment available. Depending on the spectrum of symptoms, which has proved to be very heterogeneous, management may include lifestyle modification, rehabilitation, medications, psychotherapy, acupuncture, or other non-pharmacologic interventions [21,22].

Rehabilitation strategies have been used to target cardiovascular and pulmonary symptoms and may entail the following: aerobics, endurance, resistance, strength, stretching, and motor or balance training. Patients exhibiting cognitive symptoms may also undergo cognitive rehabilitation. Thus far, the evidence of the therapeutic benefit of rehabilitation for PASC remains mixed but overall positive. Recent systematic and meta-analyses have shown improvements in functional outcomes (six-minute walk test, sit-to-stand test, and hand grip) [23-25], respiratory function (forced expiratory volume in the first second, forced vital capacity, and modified Medical Research Council dyspnea scale) [25-27], and cognitive symptoms [28].

Medication management strategies may be tailored to symptoms that are neuropsychiatric in nature, such as use of antidepressants or anxiolytics; symptoms involving fatigue or brain fog, such as use of stimulants; or symptoms involving orthostasis, such as use of fludrocortisone or midodrine. Other proposed medication options have included low-dose naltrexone, pyridostigmine, nicotinamide adenine dinucleotide (NAD+), or supplements such as N-acetylcysteine or coenzyme Q10 [29-31].

SGB treatments have been reported as beneficial in treating symptoms of PASC in small retrospective or case-series studies [31,32]. Prior initial studies comprised case reports that demonstrated significant efficacy of SGB for PASC with complete resolution of symptoms following two SGB treatments [10, 33-35]. Since these promising results demonstrated improvement in PASC symptoms and Short Form-36 Health Survey (SF-36) outcomes, interest has grown in evaluating SGB efficacy on larger scales.

More recently, Pearson et al. evaluated 41 patients who received either single or multiple SGB and found that 86% of patients reported improvement in at least one PASC symptom [36]. Fatigue, brain fog, and post-exertional malaise were the most commonly presenting symptoms, consistent with our study. Furthermore, they showed SGB provided improvement for the majority of patients with symptoms of fatigue (77%), brain fog (79%), and post-exertional malaise (74%). Impressively, the authors found all but two patients experienced persistent relief up to 12 months following SGB treatment. In comparison, we found that 55.8% of our patients reported some degree of improvement across all symptoms, and only 13.5% reported ongoing improvement from the time of SGB treatment to the time of survey completion. The large difference may partially arise from recall bias within our population. Whereas Pearson et al. reported rather rapid improvement in symptoms, we evaluated patient-reported outcomes following SGB at longer and variable time points. We found that SGB treatments sometimes resulted in improvement on the order of hours to weeks, and surveys were often completed after this amount of time. Another factor may be the variability in the severity of PASC symptoms. In our study, 86.5% of patients reported “severe” symptoms. Furthermore, patients reported a mean duration of PASC symptoms of 1.6 years prior to the first SGB treatment. This may suggest that our patient population reflected different symptom severity levels or chronicity in PASC. One interpretation is that PASC patients may respond better to earlier SGB intervention and that such responses may persist longer. Together, the relationship between symptom severity and duration and the efficacy of SGB treatment remains a critical factor that warrants further exploration.

A second important retrospective cohort study involved 27 patients in which Duricka et al. performed subgroup analysis of those who had satisfactory (durable responders, n=10), partial (partial responders, n=9), satisfactory but non-sustained (non-durable responders, n=6), or no response (non-responders, n=2) at one-month follow-up after serial SGB treatments [37]. While their sample size precluded statistical evaluation of demographic variability across these groups, the authors collected data on medical history such as body mass index, hypertension, depression, and tobacco use. Nevertheless, they showed large changes in 12 PASC symptoms, such as mean improvements in fatigue (4.7 points), problems concentrating (3.3 points), and worsened symptoms after physical activity (5.9 points), albeit with sample sizes of n = 7, 9, and 7, respectively. Compared to our changes in symptom severity scores, we found smaller improvements, i.e., three points each with fatigue and brain fog. The small sample sizes may underlie the discrepancy we see between our scores as compared to the findings by Duricka et al. However, their overall percentage of durable and non-durable responders more closely approximated our results. Durable responders at one month accounted for 37% of their patients, while 36.5% of our patients reported improvement for months or longer. Non-durable responders (less than one month) accounted for 22.2% of their patients, while 19.2% of our patients reported improvement lasting from hours to weeks. Interestingly, we were not able to replicate the improvement in the specific symptom of post-exertional malaise, which was a common symptom reported by our cohort.

Overall, our study contributes to the growing body of literature investigating the role of SGB in the management of PASC symptoms. An advantage of our retrospective study is that we evaluated the efficacy of SGBs for patients experiencing PASC symptoms for a mean (SD) duration of 2.6 (0.9) years, with the mean (SD) time to first SGB of 1.6 (0.9) years after initial diagnosis. Thus, our data may better represent the real-world patient experience from the time of initial diagnosis of PASC to first SGB and subsequent evaluation. The patient characteristics in our study were consistent with CDC data, which reported a higher prevalence of PASC in the age range of 40-59 years, in women, and in White patients. The number of queried symptoms, as well as patient-reported additional symptoms in the category of “other,” helps highlight the diverse presentation of PASC symptomatology. The majority of our patients reporting the most common symptoms (headache, fatigue, dizziness, brain fog) reported improvement of that symptom, except for post-exertional malaise. The post-SGB severity scores of these symptoms showed substantial improvement, though the sample size limits further conclusions. On a Likert scale, 46.2% of our patients reported an overall improvement. Again, it was possible for patients to report short-term improvement but answer “no change” on this scale at the time of survey completion. Overall, our results help delineate which specific symptoms appear to have a greater response and likelihood of response, help define the durability of improvement, and also report patients' overall impression of change.

Of note, throughout our experience during this study, we noted SGB treatments have sometimes been cost-prohibitive due to a lack of insurance coverage and/or high out-of-pocket expenses. The expense of treatment may preclude patients, particularly those who belong to marginalized groups, from pursuing SGB treatment for PASC symptoms, perpetuating health disparities already intrinsically tied to COVID-19 infection. As multiple studies, including our own, have demonstrated the efficacy of SGB for managing PASC symptoms, in addition to the mixed efficacy and potential adverse effects of medication management, consideration should be made to increase accessibility for this potentially helpful procedure. Continued research would clearly be helpful in this regard.

We identified several significant limitations of the study. Notably, our study lacked adequate power to quantitatively analyze the data. Consequently, we can only infer from the descriptive data the efficacy of SGB performed at our institution. Patient self-reported assessments were performed retrospectively; thus, significant recall bias may have affected differences between patients’ improvement shortly after the SGB procedure as compared to the time delay to survey completion. Nevertheless, this study may provide a realistic picture of patients’ assessment of improvement, in other words, whether patients reported long-term significant benefit from the procedure. An additional limitation reflects the lack of standardization for patient follow-up and assessment. The vast majority of our patients (approximately 90%) received more than one SGB. Therefore, more frequent assessments can better characterize the impact of laterality and multiple injections on PASC symptoms. Another potential source of variability may be proceduralist technique and differences in injectate (addition of corticosteroid with local anesthetic). We did not have a control group, so we cannot directly assess for confounding variables, such as the degree of placebo effect. Future prospective cohort studies may incorporate a control group, randomization, blinding, larger sample sizes, and standardized evaluation times to better characterize the efficacy of SGB treatments on PASC symptomatology.

## Conclusions

The results from our retrospective study suggest reductions in the severity of many PASC symptoms, consistent with previous studies. There was a high level of variability in the duration of symptom improvement, with substantial heterogeneity in types of reported symptoms. Thus, further high-quality randomized clinical trials are warranted to investigate SGBs for the effective treatment of PASC.

## Figures and Tables

**Figure 2:**
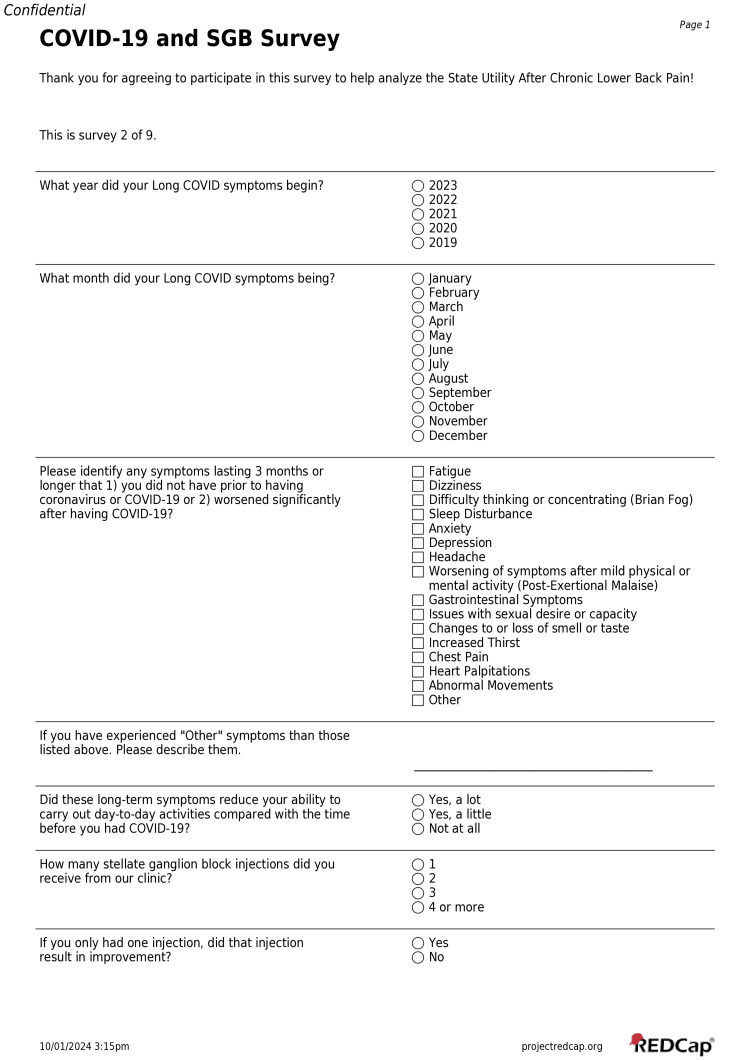
Page 1 of the survey

**Figure 3:**
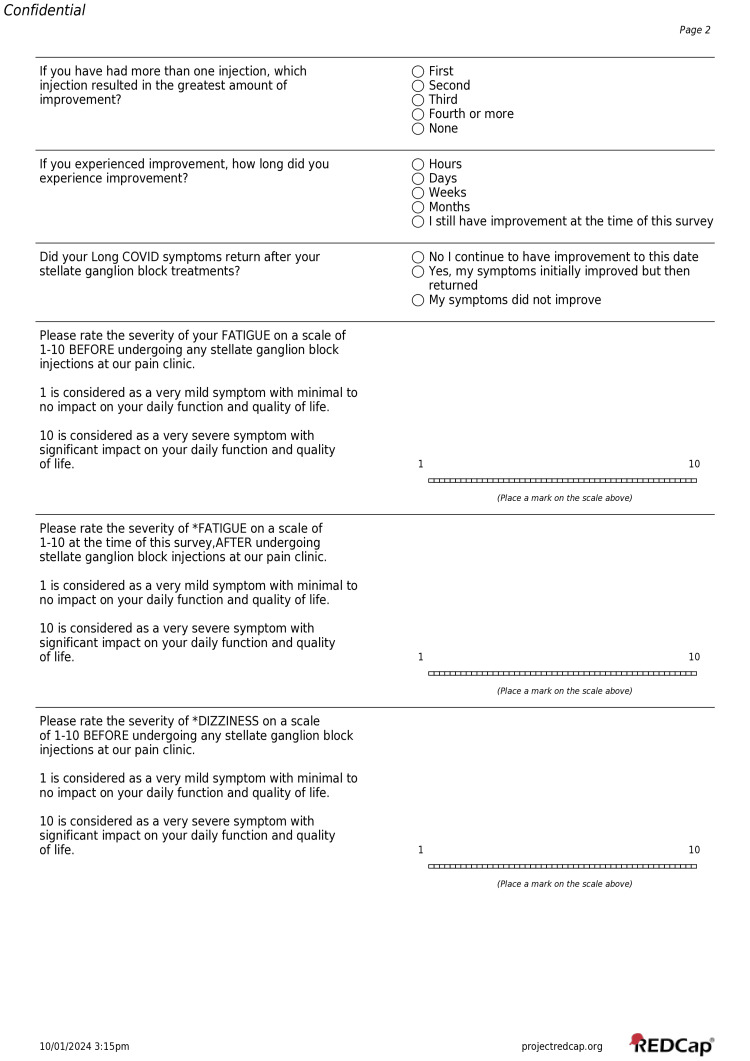
Page 2 of the survey

**Figure 4:**
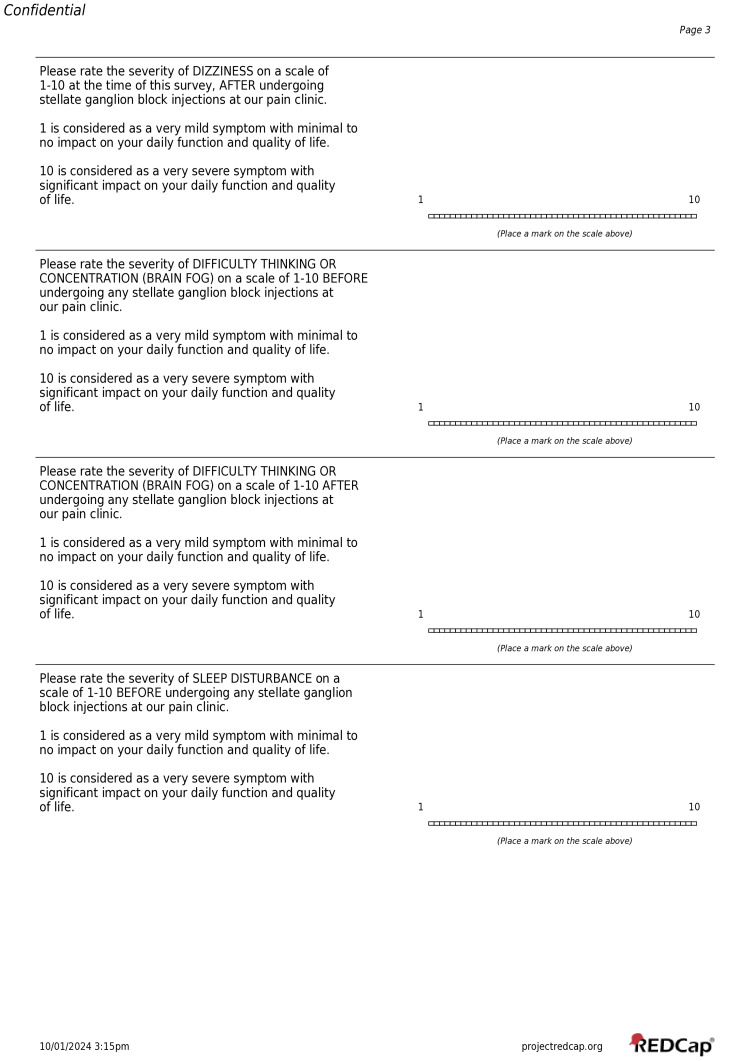
Page 3 of the survey

**Figure 5:**
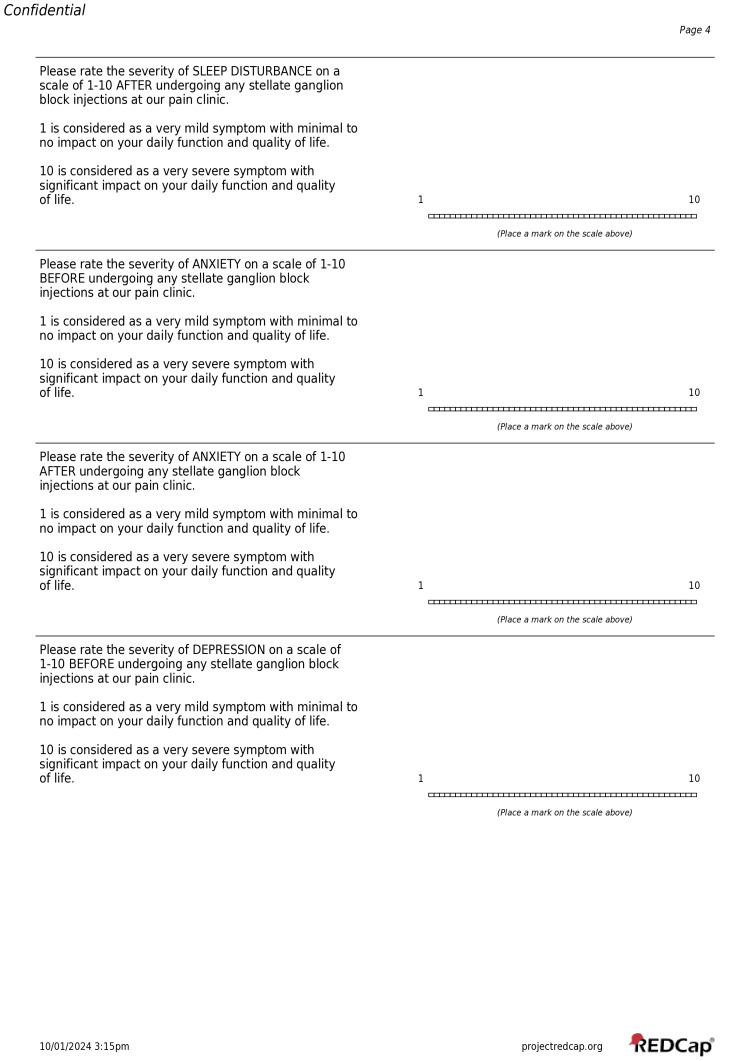
Page 4 of the survey

**Figure 6:**
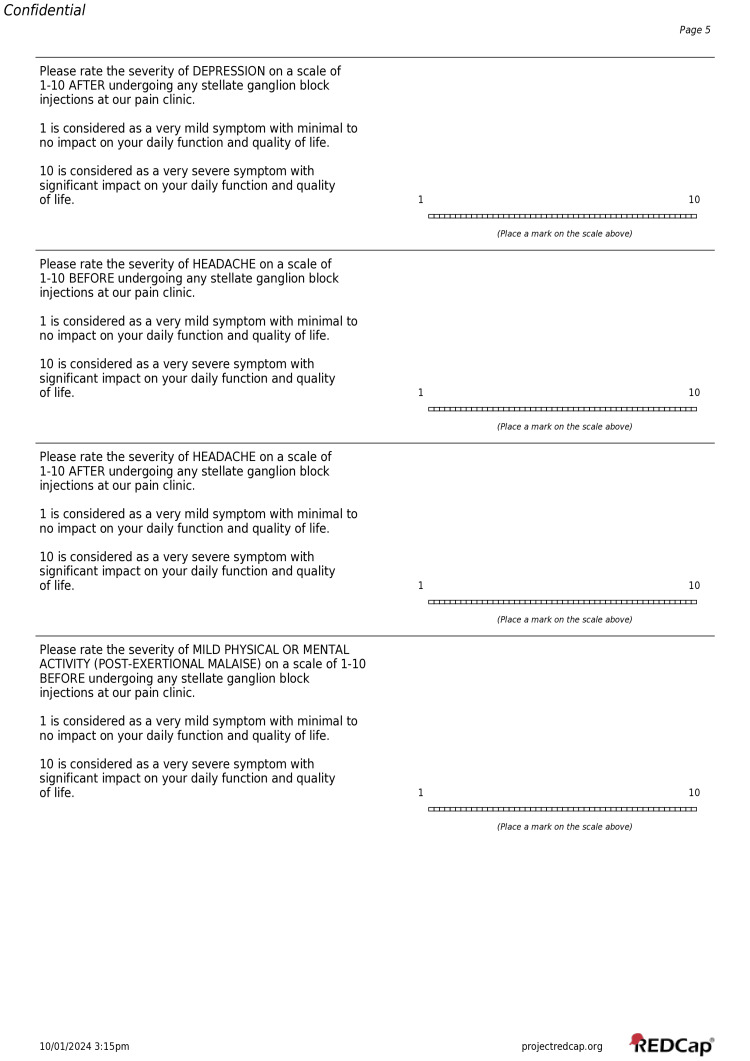
Page 5 of the survey

**Figure 7:**
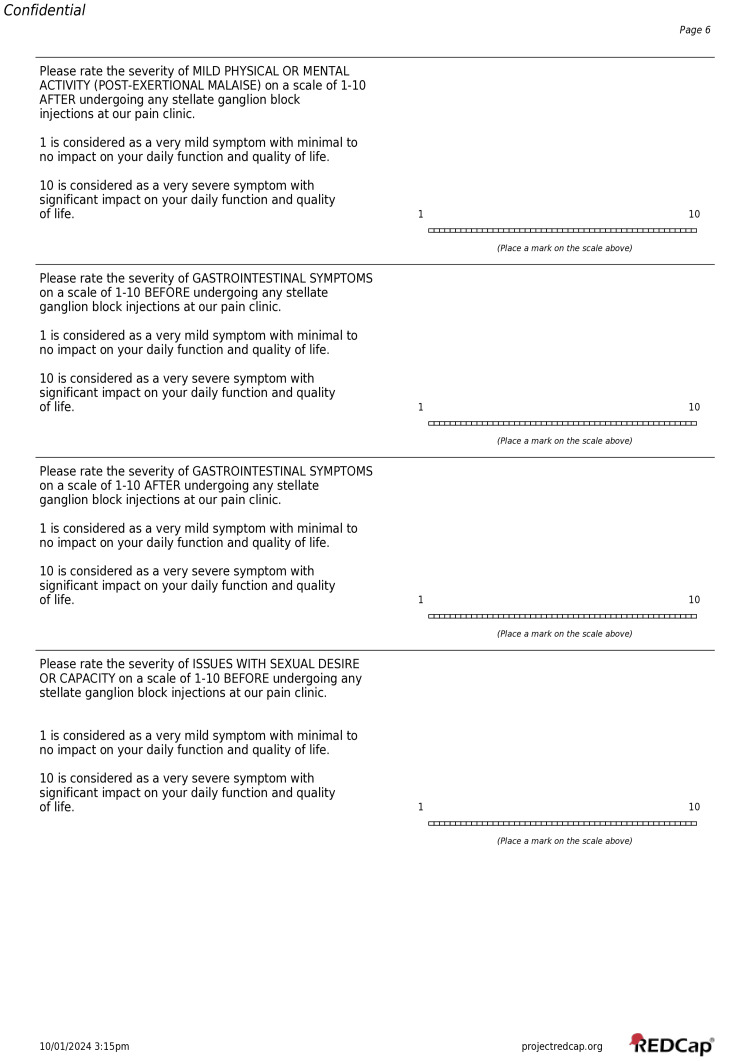
Page 6 of the survey

**Figure 8:**
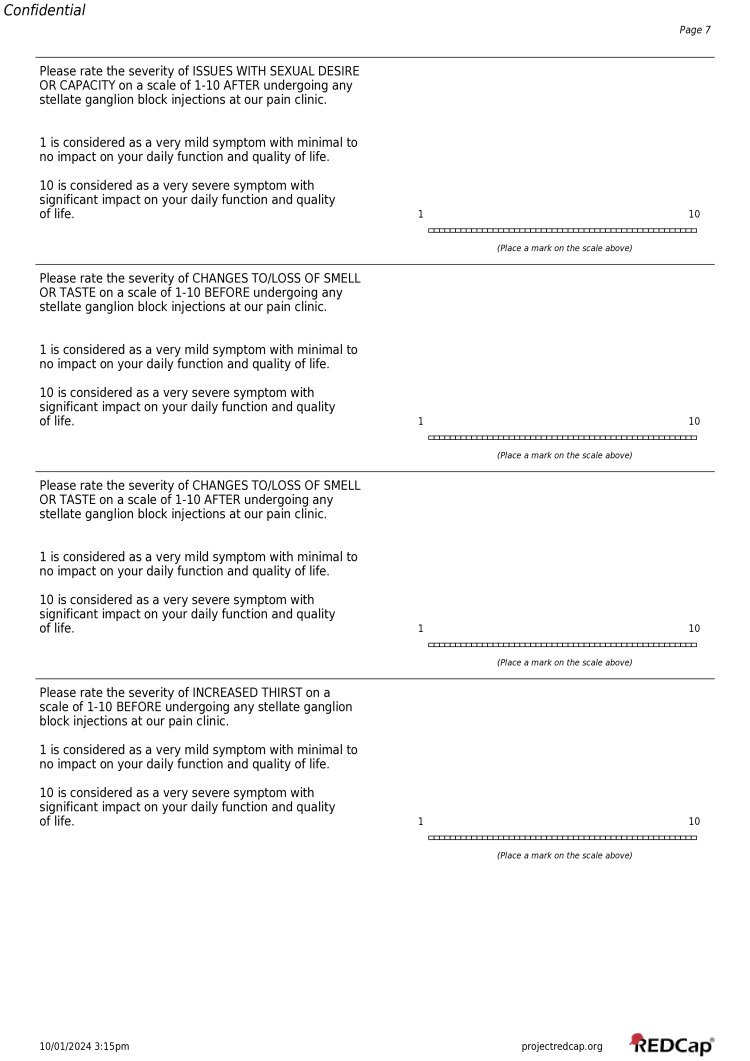
Page 7 of the survey

**Figure 9:**
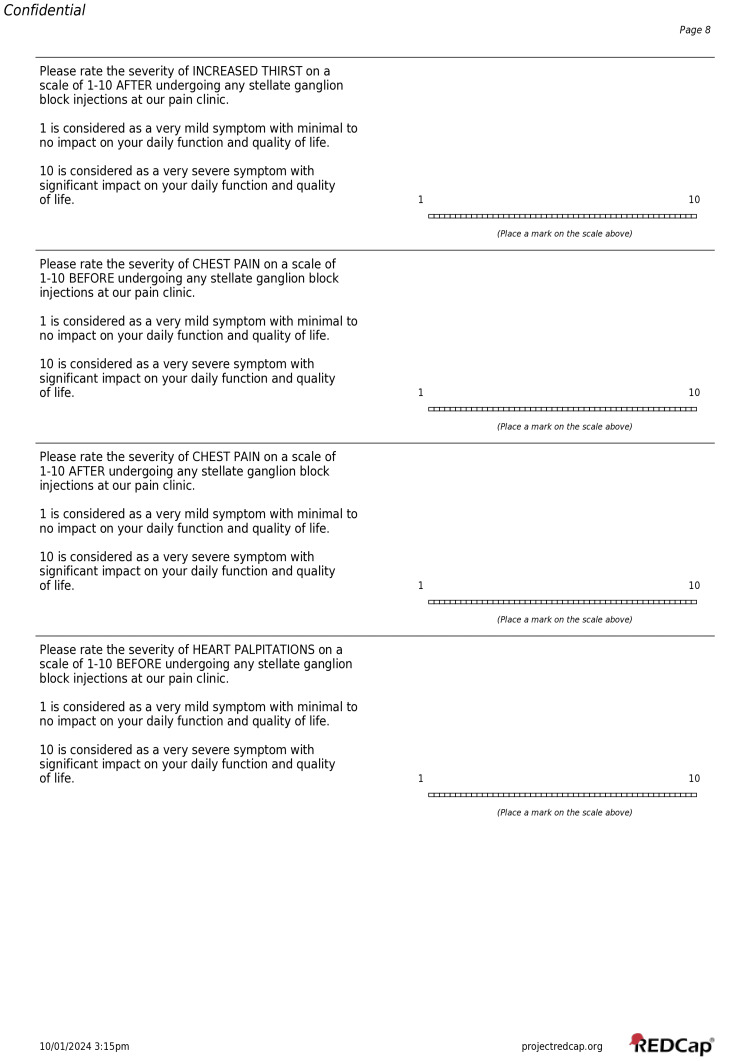
Page 8 of the survey

**Figure 10:**
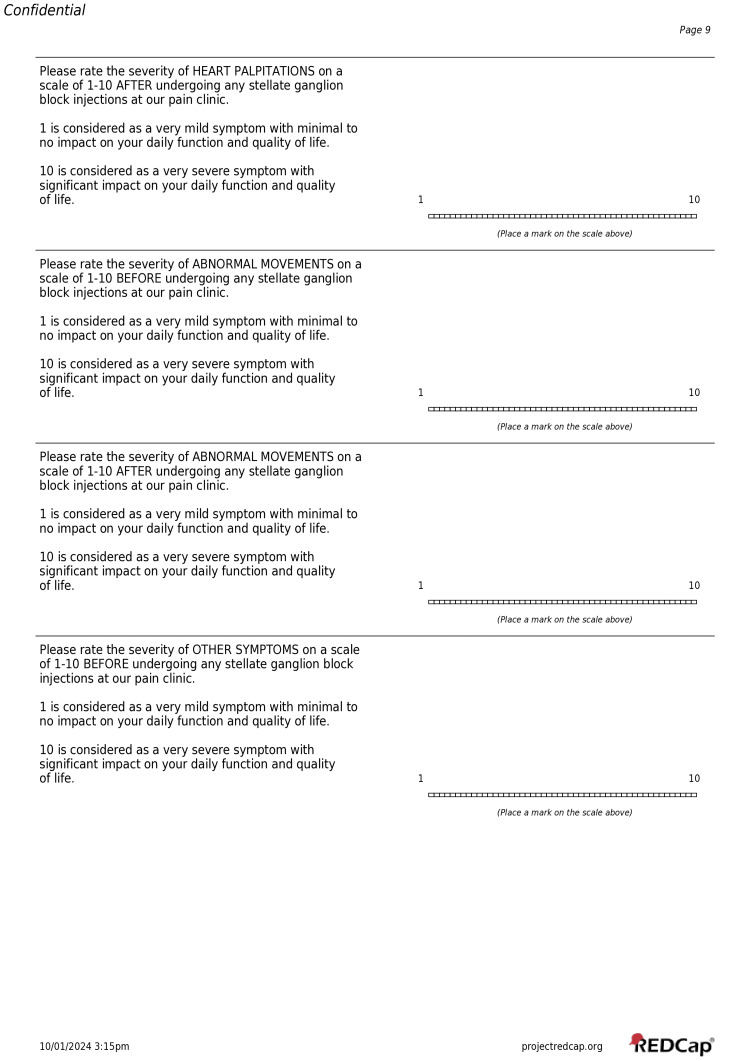
Page 9 of the survey

**Figure 11:**
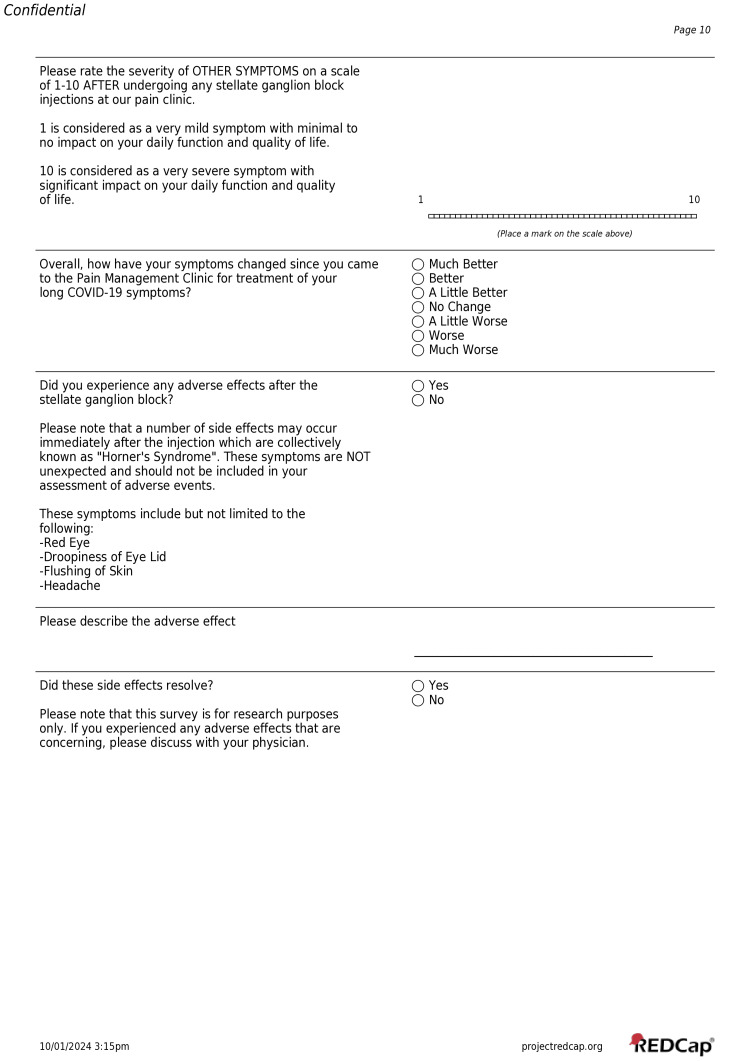
Page 10 of the survey

**Figure 12:**
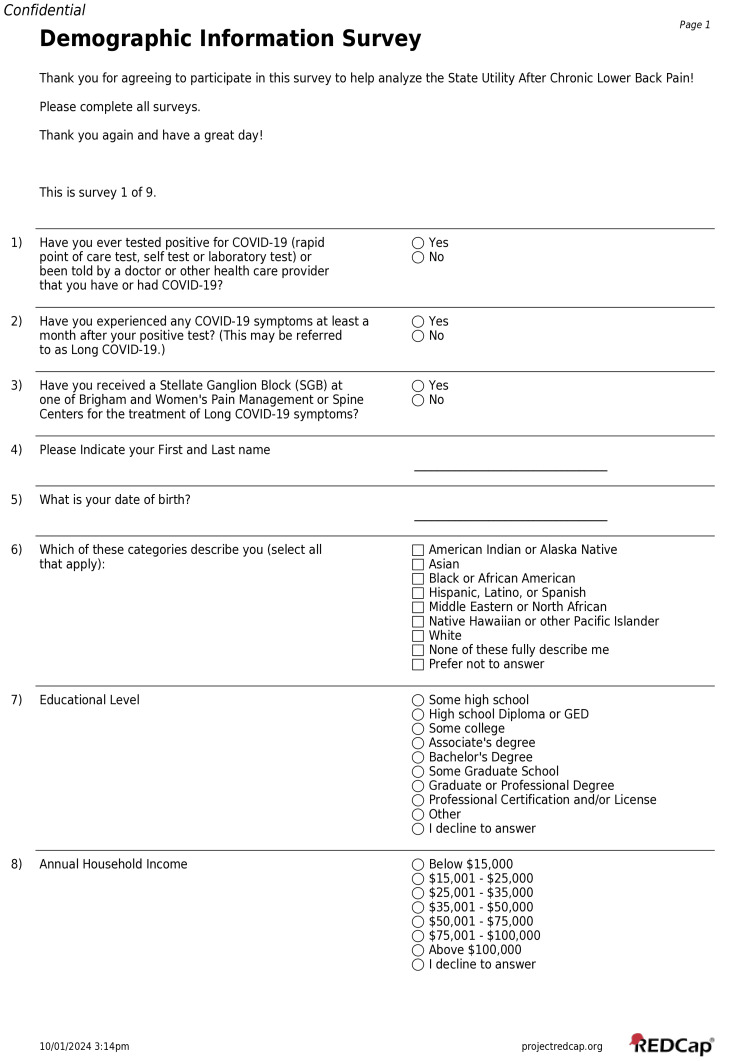
Demographic information

## Appendices

Appendix A

Electronic Survey Sent to Patients

Appendix B

**Table 7:** Patient-reported symptoms categorized as “Other” Severity was reported on a three-point Likert scale.

Symptom	Frequency	Severity
Dysautonomia	4	Severe
Myalgia	3	Severe
Orthostasis	3	Severe
Shortness of breath	3	Severe
Arthralgia	2	Severe
Tinnitus	2	Severe
Dysesthesia	1	Severe
Dysgeusia	1	Severe
Hair loss	1	Severe
Nausea	1	Severe
Parosmia	1	Severe
Unsteadiness	1	Severe
